# Evaluation of cardiac output by 5 arterial pulse contour techniques using trend interchangeability method

**DOI:** 10.1097/MD.0000000000003530

**Published:** 2016-06-24

**Authors:** Marc-Olivier Fischer, Momar Diouf, Robert B.P. de Wilde, Hervé Dupont, Jean-Luc Hanouz, Emmanuel Lorne

**Affiliations:** a Pôle Réanimations Anesthésie SAMU/SMUR, CHU de Caen; b EA 4650, Université de Caen Normandie, Esplanade de la Paix, Caen; c Department of Biostatistics and Clinical Research, Amiens University Hospital, Place Victor Pauchet, Amiens, France; d Department of Intensive Care, Leiden University Medical Center, RC Leiden, The Netherlands; e Anesthesiology and Critical Care Department, Amiens University Hospital, Amiens; f INSERM U 1088, Jules Vernes University of Picardy, Centre Universitaire de Recherche en Santé (CURS). Chemin du Thil, Amiens Cedex, France.

**Keywords:** anesthesiology, cardiac output, interchangeability, measurements

## Abstract

Cardiac output measurement with pulse contour analysis is a continuous, mini-invasive, operator-independent, widely used, and cost-effective technique, which could be helpful to assess changes in cardiac output. The 4-quadrant plot and the polar plot have been described to compare the changes between 2 measurements performed under different conditions, and the direction of change by using different methods of measurements. However, the 4-quadrant plot and the polar plot present a number of limitations, with a risk of misinterpretation in routine clinical practice. We describe a new trend interchangeability method designed to objectively define the interchangeability of each change of a variable. Using the repeatability of the reference method, we classified each change as either uninterpretable or interpretable and then as either noninterchangeable, in the gray zone or interchangeable. An interchangeability rate can then be calculated by the number of interchangeable changes divided by the total number of interpretable changes. In this observational study, we used this objective method to assess cardiac output changes with 5 arterial pulse contour techniques (Wesseling's method, LiDCO, PiCCO, Hemac method, and Modelflow) in comparison with bolus thermodilution technique as reference method in 24 cardiac surgery patients. A total of 172 cardiac output variations were available from the 199 data points: 88 (51%) were uninterpretable, according to the first step of the method. The second step of the method, based on the 84 (49%) interpretable variations, showed that only 18 (21%) to 30 (36%) variations were interchangeable regardless of the technique used. None of pulse contour cardiac output technique could be interchangeable with bolus thermodilution to assess changes in cardiac output using the trend interchangeability method in cardiac surgery patients. Future studies may consider using this method to assess interchangeability of changes between different methods of measurements.

## Abstract 2

Supplemental Digital Content is available in the text

## 1 Introduction

Cardiac output (CO) monitoring during the perioperative period could decrease both morbidity and length of hospital stay and has been recommended in high-risk surgical patients.^[1–4]^ Theoretically, the ideal CO monitor should not only accurately measure CO, but also guide hemodynamic optimization by assessing fluid responsiveness during therapeutic maneuvers.^[5]^ At the bedside, the real-time tracking of the direction of changes in CO could be more useful than the ability to give a highly accurate single measurement under stable hemodynamic conditions.^[6]^ CO measurement with pulse contour analysis is a continuous, mini-invasive, operator-independent, widely used, and cost-effective technique, which could be helpful to assess changes in CO.

The simplest method to describe trending is to plot the test and reference methods (RMs) together against time, but no objective method is available for the interpretation of data from single or multiple patients. The 4-quadrant plot was subsequently described to objectively measure trending by using the concordance rate, according to the percentage of concordant data points in terms of the direction of change of the value between the 2 methods.^[7,8]^ However, the value of the changes between the test and RMs can be very different, but may be concordant if the changes observed with the 2 methods are both in the same direction, which can lead to unreliable physician decisions. Polar plots have recently be proposed to address this issue, by converting data to polar coordinates.^[9,10]^ However, this method is associated with a high risk of misclassification when 2 methods of measurement indicate changes in opposite directions.^[11]^ Moreover, this method does not take into account the repeatability of the RM and the polar limits of agreement are not calculated a priori, thereby preventing objective interpretation.

In this work, we describe the limitations of previous methods and we showed the interest of a new and simple method using the repeatability of the RM to objectively calculate the trend interchangeability rate between different methods of measurement. We used this objective method to assess CO changes with 5 arterial pulse contour techniques (Wesseling's method, LiDCO, PiCCO, Hemac method, and Modelflow) in comparison with bolus thermodilution technique as RM. We tested the hypothesis that arterial pulse contour techniques could be interchangeable with bolus thermodilution to assess CO changes accurately.

## 2 Materials and methods

### 2.1 Patients

After approval from the local ethics committee (hospital ethics committee of Leiden University Medical Center) written informed consent was obtained for all patients the day before surgery. Inclusion criteria were coronary surgery with cardiopulmonary bypass, and without congestive heart failure or concomitant heart valve disease. The study was conducted in accordance with the STROBE Statement.^[12]^

### 2.2 Study design

The study design was previously published.^[13]^ Briefly, 24 consecutive patients were included during cardiac surgery in Leiden University Medical Center (The Netherlands) from February 1992 to June 1996. CO measurements by 5 different arterial pulse contour techniques using arterial catheter, usually used in cardiac surgery patients (Wesseling's method, LiDCO, PiCCO, Hemac method, and Modelflow), were simultaneously recorded during 4-bolus pulmonary artery thermodilution as RM. The measurements were conducted during different predefined times: 3 minutes after the induction of anesthesia, immediately after sternotomy, after opening of the pericardium, just before and just after cardiopulmonary bypass, after sternal fixation, after the completion of the surgery, and after changes in drug dose.

### 2.3 Descriptions and limitations of previous methods used to assess the trend of measurements

#### 2.3.1 Four-quadrant plots

Four-quadrant plots, first described for CO measurements,^[7]^ demonstrates changes between the test and RMs used to measure CO. The plot is divided into 4 quadrants around the *X*- and *Y*-axes that intersect at the center (0,0). After exclusion of points considered to represent clinically insignificant changes or the imprecision of the method (defined as the central zone), concordance analysis is performed by counting the number of remaining data points situated within the 2 quadrants of agreement (upper right and lower left quadrants). A concordance rate is then calculated and a line of identity *Y* = *X* is depicted.

The limitations of this method are (i) calculation of exclusion of the central zone has not been standardized (exclusion criteria based on absolute values are situated below an arbitrary limit or below a percentage change, e.g., 10% or 15%),^[8]^ (ii) the concordance rate is calculated by means of an imprecise method (e.g., a plot could be classified as concordant if a change was recorded as 1000% for the RM and 1% for the test method, TM), and (iii) the absence of guidelines for correct interpretation of the concordance rate.

#### 2.3.2 Polar plots

This more recently described method converts a paired series of measurements [readings from the RM (*X*-axis) and TM (*Y*-axis) on an *X*-*Y* plot] to polar coordinates. The angle made with the line of identity *Y* = *X* is used to calculate each vector for each change of measurement.^[9,10]^ An angular bias ± 5° or less and radial limits of agreement ± 30° or less are proposed.

The limitations of this method are (i) the central exclusion zone is not objectively calculated (an arbitrary value of 0.5 L/min for CO was used),^[10]^ (ii) the 30° limits are based on incomplete results using data extracted by software^[10]^ from the original data of the previous study conducted in 24 postcardiac surgical patients,^[13]^ and these 30° limits were subjective, (iii) limits of agreements were used by the authors, and no trend interchangeability rate was calculated, and (iv) the exclusion zone of the polar plot excludes from the analysis all changes that have similar absolute values, but in opposite directions, which corresponds to the most discordant measurements.^[11]^

### 2.4 Proposed method to assess interchangeability of changes of measurements

**Figure 1:**
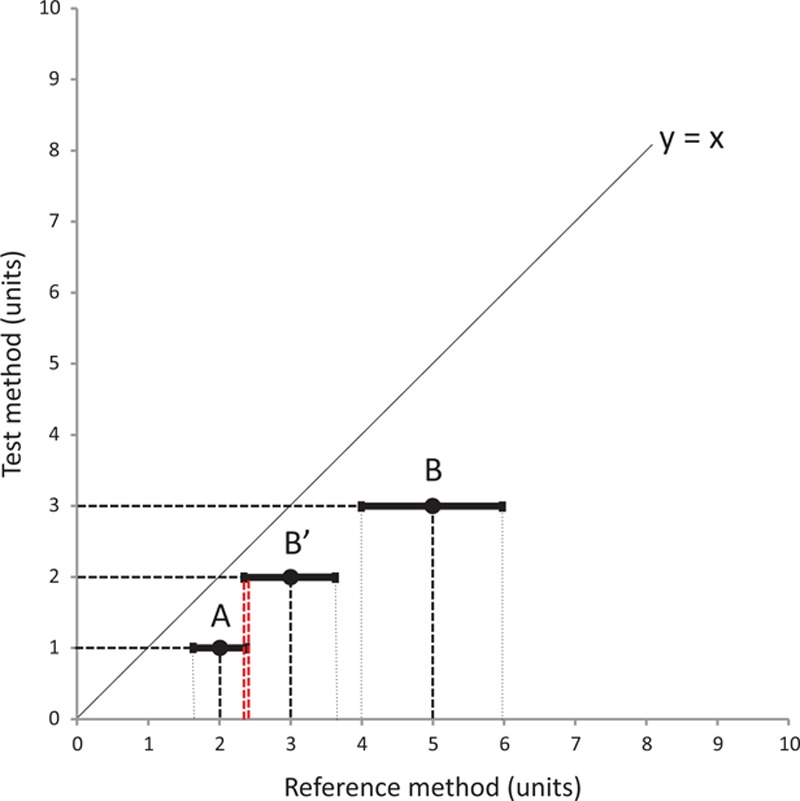
First step of the interchangeability method to assess changes in measurements between 2 methods of measurement. For a repeatability of 20% for this example, point A could change to either point B (interpretable variation) or point B’ (uninterpretable variation). The repeatability of measurement, indicated by the solid segment on both sides of each point, overlaps for points A and B’.

**Figure 3:**
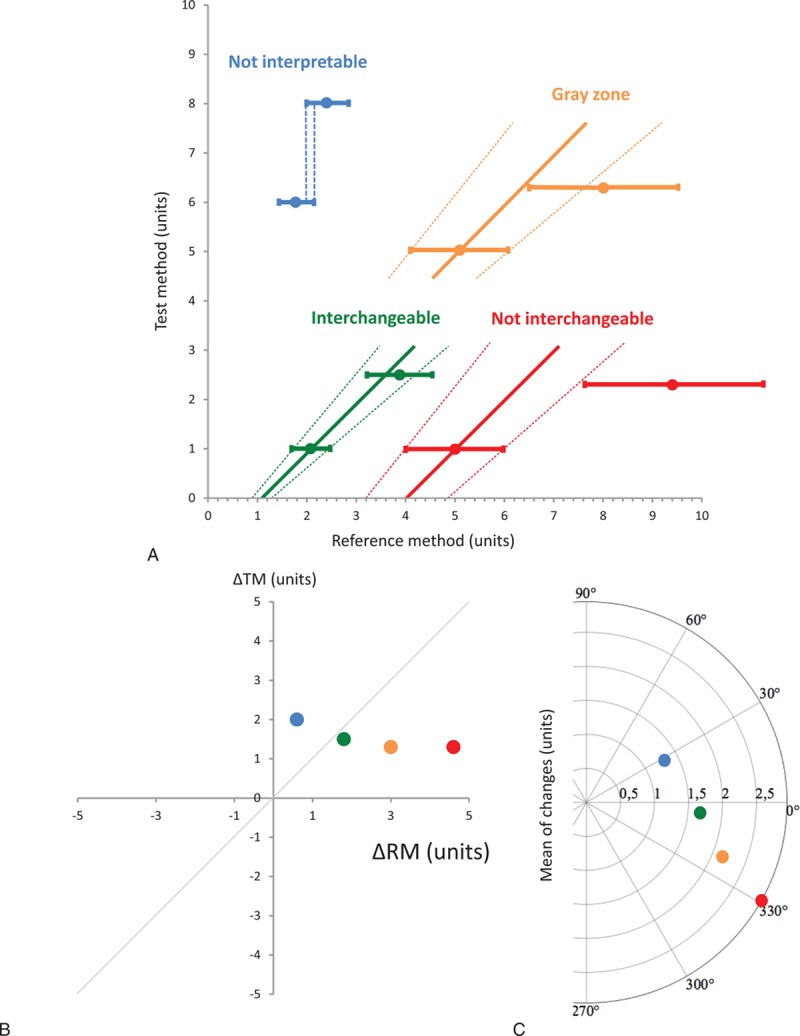
Didactic color-coded figure showing each possible situation when analyzing changes in measurements between 2 methods: uninterpretable (blue), noninterchangeable (red), in the gray zone of interpretation (orange), and interchangeable (green) (A). The last 3 points are shown with their interchangeability line and the limits of interchangeability lines. The same variations are depicted in the graphical representation for each situation in a 4-quadrant plot (B) and a polar plot (C). ΔRM = changes in reference method, ΔTM = changes in test method.

According to the guidelines for reporting reliability and agreement studies,^[14]^ we propose a new method to assess the interchangeability of the trends observed with 2 methods of measurement. The first step of the proposed method is to determine whether or not each variation is interpretable. We postulate that a change can only be interpretable when the confidences intervals of the reference values (reference value ± reference value multiplied with the repeatability coefficient) of the 2 measurements, do not overlap. A change between points A and B would then be interpretable (A and B with nonoverlapping confidences intervals) or uninterpretable (A and B’ with overlapping confidences intervals), as described in Fig. 1 or Fig. 3A.

The second step consists of determining whether each interpretable change is interchangeable, situated in an uncertain interchangeable zone (gray zone), or not interchangeable.

**Figure 2:**
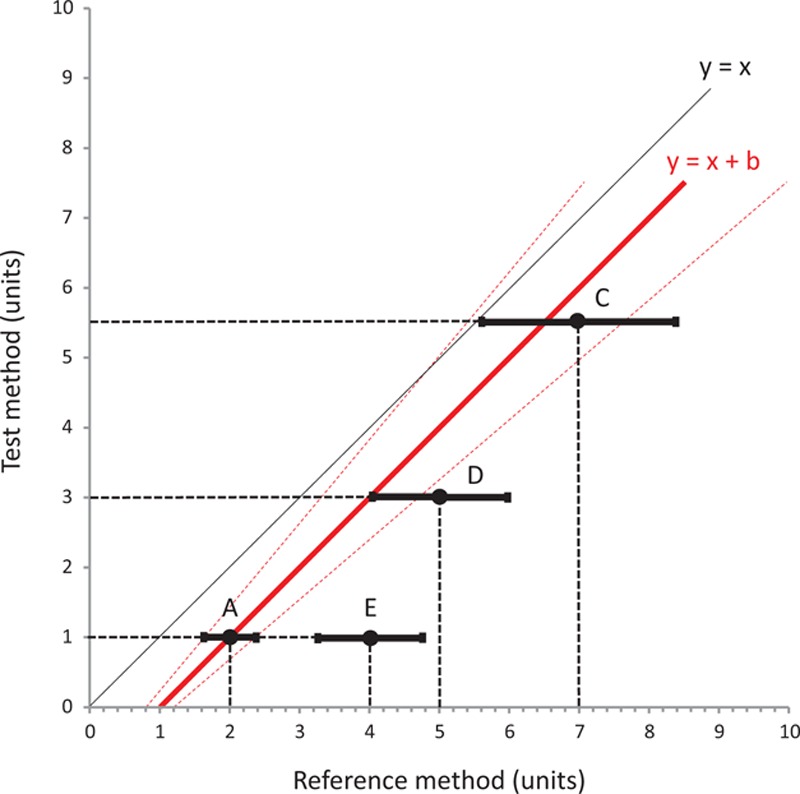
Second step of the interchangeability method to assess changes in measurements between 2 methods of measurement. Initial point A changes to point C, which is situated inside the zone between the 2 interchangeability lines (dotted red lines, defined as [(*X* = *Y*(1+*R*)+(1+*R*) (RM_1_−TM_1_)) and (*X* = *Y*(1−*R*)+(1+*R*) (RM_1_−TM_1_)]), D if only the repeatability overlaps with the same zone, or E if neither the point itself nor its repeatability interval overlap with the same zone. *R* = repeatability, RM_1_ = first value measured with the reference method, TM_1_ = first value measured with the test method.

We also postulate that a change can be considered to be interchangeable with another change if the second pair of measurements lies within a predicted precision interval of the RM. This interval is derived from the predicted line of identity of the RM of the first pair of measurements and the repeatability coefficient of the RM (Fig. 2). Repeatability (*R*) has been previously defined as follows:  
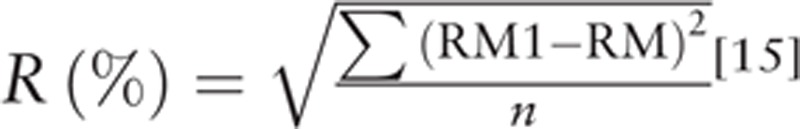
^[15]^. The repeatability coefficient (RC) can be defined as  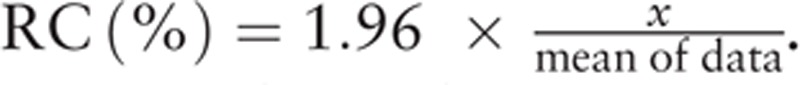.

For a change between 2 points A and B measured by the RM, each point can be defined by both its value and its repeatability as follows: A ± (A × repeatability coefficient of the RM) will change to B ± (B × repeatability coefficient of the RM). As described in Fig. 2, a variation between points A and C can be considered to be interchangeable if point C is situated inside the confidence interval of interchangeable changes initiated from the first point. These 2 lines are defined by the equations [(*X* = *Y*(1+RC)+(1+RC) (A_RM_−A_TM_)) and (*X* = *Y*(1−RC)+(1+RC) (A_RM_−A_TM_)], where A_RM_ is the value for point A using the RM and A_TM_ is the value for point A using the TM (dotted red lines represented in Fig. 2). The interchangeability of a variation between A and D can be considered to be uncertain when the interval of precision of point D intersects 1 of the 2 lines of confidence intervals of the interchangeable change, but does not contain point D itself. Finally, the change can be considered to be noninterchangeable when neither E nor its repeatability are situated in the zone previously described. Figure 3A summarizes the 4 possible situations. These changes can also be represented graphically with the same color code in a 4-quadrant plot (Fig. 3B) and in a polar plot (Fig. 3C).

According to the situation of each point, as described earlier, the trend interchangeability rate expressed as a percentage can then be calculated for the whole set of interpretable measurements.

### 2.5 Dataset

We simulated 300 variations of measurements for 150 patients (3 values and 2 changes for each patient). The data points were obtained in 3 ranges of values (2.5, 3.5, and 5 units) using R Software version 3.1.0 (R Core Team, R Foundation for Statistical Computing, Vienna, Austria). One hundred values were simulated for each of the 3 ranges using 3 multivariate normal distributions, as follows:  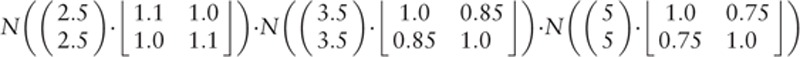. The variance-covariance matrices were chosen so that the variance of the measurements decreased with their absolute values.

The R script is available in Appendix 1, and the simulated data are available in Appendix 2. We then applied the proposed method to the complete original data published by de Wilde et al.^[13]^ This study compared CO measurements by 5 different arterial pulse contour techniques (Wesseling's method, LiDCO, PiCCO, Hemac method, and Modelflow) in comparison with the 4-bolus pulmonary artery thermodilution as RM.^[13]^

### 2.6 Primary endpoint

The primary endpoint of the present study was to calculate the trend interchangeability rate between 5 different arterial pulse contour techniques (Wesseling's method, LiDCO, PiCCO, Hemac method, and Modelflow) of CO measurements in comparison with pulmonary artery thermodilution as RM.

### 2.7 Simplified algorithm of the trend interchangeability method to compare changes using 2 methods of measurements

**Figure 4:**
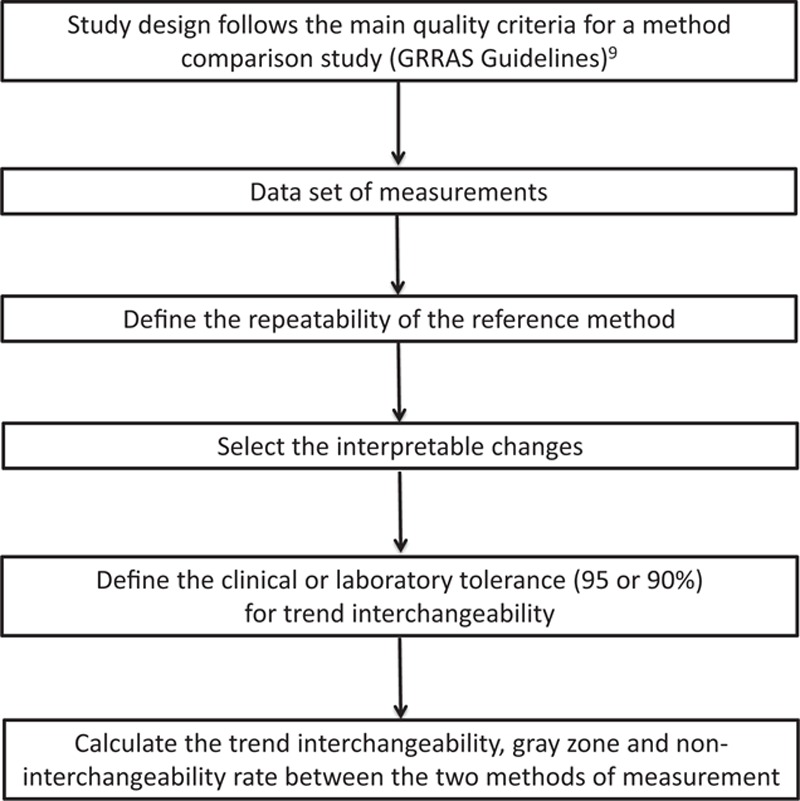
Simplified algorithm for the trend interchangeability method to assess changes in measurements between 2 methods of measurement.

Figure 4 shows the successive steps to compare changes of measurements between 2 methods.

### 2.8 Statistical analyses

The number of simulated data was empirically set at 300 before starting the analysis. Trend interchangeability (i.e., the proportion of interchangeable variations) was then calculated, expressed as number (percentage), and was considered to be excellent (≥95%), good (≥ 90%), poor (75%–90%), or not clinically relevant (<75%) according to its value. First data (used to calibrate the 5 pulse contour methods), and missing data were not used. A chi-square test was performed to compare the interchangeability rate between the 5 tested devices. A *P* value <0.05 was considered to be statistically significant and all *P* values were 2-tailed. Statistical analyses were performed with Excel version 14.4.8 (Microsoft Corporation, Redmond, Washington), and R software version 3.0.1 (www.r-project.org). Polar plot figures were performed with Veusz software (GitHub, San Francisco, CA).

## 3 Results

### 3.1 Simulated data

**Figure 5:**
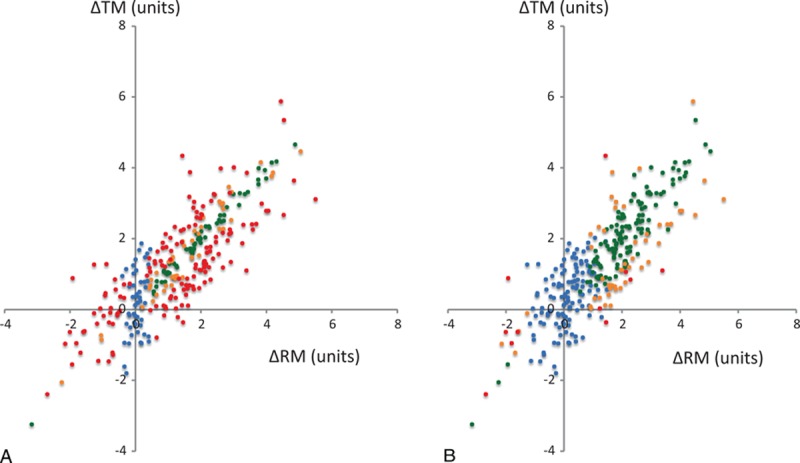
Simulated data in a 4-quadrant graphical representation using reference method repeatabilities of 5% (A) and 20% (B). A specific color is applied to each change: uninterpretable (blue), noninterchangeable (red), in the gray zone of interpretation (orange), and interchangeable (green). ΔRM = changes in reference method, ΔTM = changes in test method.

Three hundred simulated data points were analyzed. A wide distribution of measurements was observed, ranging from 0.01 to 8.12 units for the RM and from −0.22 to 7.80 units for the TM; the mean value for both methods was 3.67 units. The changes of measurements for the RM and TM were −3.17 to 5.50 units and −3.24 to 5.58 units, respectively. When the repeatability coefficient of the RM was set at 5%, 50 (16%) changes were uninterpretable, 149 (50%) were noninterchangeable, 45 (15%) were situated in the gray zone, and 56 (19%) were interchangeable. In contrast, when the repeatability coefficient of the RM was set at 20%, 117 (39%) changes were uninterpretable, 11 (4%) were noninterchangeable, 49 (16%) were situated in the gray zone, and 123 (41%) were interchangeable. Graphical representation using the previous color code is presented for *R* = 5% (Fig. 5A) and *R* = 20% (Fig. 5B), respectively. According to the previous definition, the trend interchangeability rate was then calculated as 56/250 (22%) for *R* = 5%, and 123/183 (67%) for *R* = 20%. All data and calculations are presented in Appendix 2.

### 3.2 Evaluation of cardiac output changes by 5 pulse contour techniques using the trend interchangeability method

**Figure 6:**
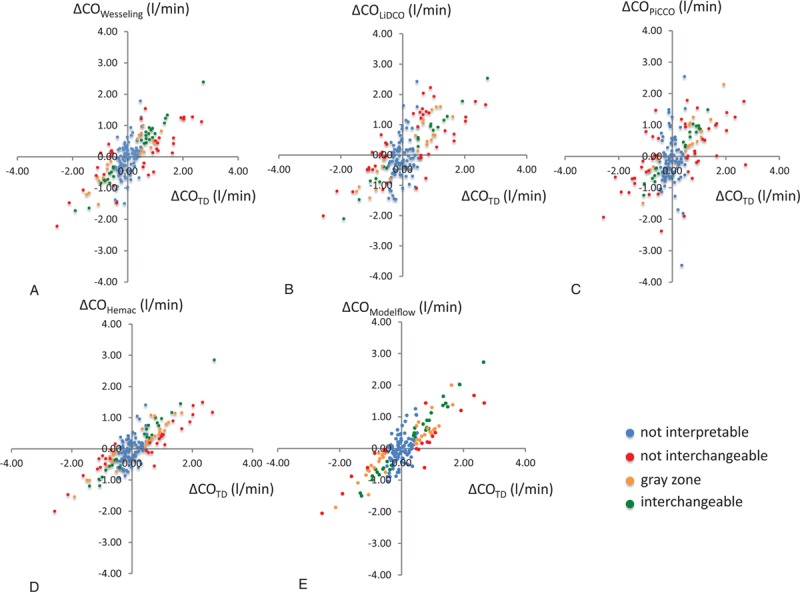
Graphical 4-quadrant plot representation of the original data previously published by de Wilde, comparing cardiac output measurement with thermodilution and 5 arterial pulse contour devices: Wesseling's method (A) LiDCO (B), PiCCO (C), Hemac method (D), and Modelflow (E), (N = 172). A specific color is applied to each change: uninterpretable (blue), non-interchangeable (red), in the gray zone of interpretation (orange), and interchangeable (green).

**Figure 7:**
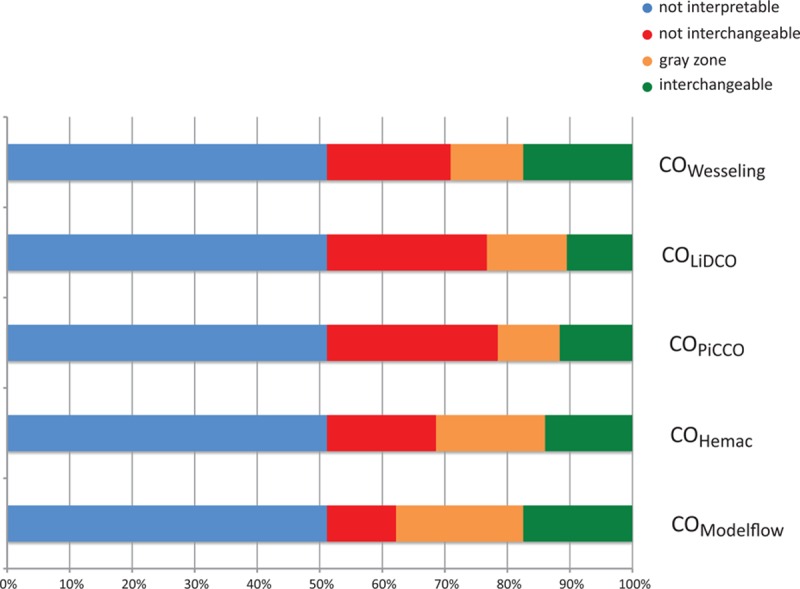
Simplified representation of the original data previously published by de Wilde, comparing cardiac output measurement with thermodilution and 5 arterial pulse contour devices: Wesseling's method (Fig. 6A) LiDCO (Fig. 6B), PiCCO (Fig. 6C), Hemac (Fig. 6D), and Modelflow (Fig. 6E; *N* = 172). A specific color is applied to each change: uninterpretable (blue), noninterchangeable (red), in the gray zone of interpretation (orange), and interchangeable (green).

Original data published by de Wilde et al.^[13]^ and used by Critchley et al.^[10]^ to define the polar plot method were reanalyzed using the new method. According to de Wilde's methods,^[13]^ we used a repeatability of 5% for the 4-cold-bolus pulmonary artery thermodilution technique.^[16]^ A total of 172 CO variations were available from the 199 data points from 24 included patients: 88 (51%) were uninterpretable, according to the first step of the method. The second step of the method, based on the 84 (49%) interpretable variations, showed that only 18/84 (21%) to 30/84 (36%) variations were interchangeable regardless of the technique used. The results obtained with the 4-quadrant plot are shown in Fig. 6 and the results obtained with the simplified method are shown in Fig. 7. No statistical difference was observed between arterial pulse contour techniques (*P* = 0.130). Data are available in Appendix 3.

## 4 Discussion

The main findings of the present study conduct in cardiac surgery setting are (i) more than half of the recorded changes were considered to be uninterpretable and (ii) interpretable changes are weakly interchangeable with bolus thermodilution, whatever the pulse contour method used.

The advantages of the trend interchangeability method are that it takes into account the interchangeability of each change and objectively defines the trend interchangeability rate. The statistical analysis associated with this method comprises 2 steps: (i) define whether or not each change is interpretable, according to the repeatability of the measurements of the RM and (ii) define the interchangeability status for each change and calculate the interchangeability, noninterchangeability, and gray zone rates for the overall interpretable change.

Some authors consider that reliable real-time tracking of the direction of changes of measurements may be more important than the ability of the device to deliver a highly accurate single measurement under stable conditions.^[17]^ However, the available statistical analysis appears to be imprecise, resulting in a risk of misinterpretation.^[11]^ The 4-quadrant plot was primarily proposed to provide a simple description of the ability of the tested device to track the direction of change obtained with the RM.^[7,8]^ However, this method did not take into account the magnitude of the changes between the 2 methods of measurements. More recently, the polar plot method has tried to resolve this limitation by using an angular sector for each change,^[9,10]^ but it is difficult to interpret and some changes may be misclassified.^[11]^ We propose a new method, which classifies each change as either uninterpretable, noninterchangeable, in a gray zone, or interchangeable. An interchangeability percentage can therefore be calculated. Figure 3 shows that 4 different changes included in both the concordance quadrant and the radial limits of agreement (±30°) could be reclassified as uninterpretable (although the variation was more than 1 unit, as frequently described), noninterchangeable, in the gray zone, and finally only 1 of the 4 changes was classified as interchangeable.

One additional explanation for misclassification of the polar plot is that interchangeable changes were not included in a constant angular sector, as we have shown that interchangeable changes between the RM and the adjacent limit of interchangeability are not dependent on a constant angle (see Appendix 4). The graphical representation (Appendix 4) showed that the greater the difference between the 2 points measured with the RM, the smaller was the angular sector.

Our method is based on the repeatability of measurements with the RM. In Fig. 5, based on simulated data, the results of interchangeability are markedly different when 2 different repeatability coefficients are used. Our results suggest that when repeatability is low, the number of interpretable changes is high, but the number of interchangeable changes was low. In contrast, when the repeatability is high, fewer changes are interpretable, but more interchangeable changes are observed. This last point emphasizes the importance of a reliable measurement of the repeatability. For the interpretation of the de Wilde data, we used a repeatability at 5%, as recently described for the 4-bolus thermodilution method for measurement of CO.^[16]^ However, large independent multicenter studies in large patient populations are mandatory to determine the repeatability of the RM, because the choice of repeatability could change the interpretation of the results. Moreover, if the device has a high level of repeatability, it could be difficult for the device to detect a change in measurement, which could partially explain certain negative results from clinical utility/outcome studies using monitoring devices.

Reinterpretation of de Wilde's dataset emphasizes the large proportion of uninterpretable results, as more than half of the recorded changes were considered to be uninterpretable. Future validation studies will need to include a large cohort of patients to increase the number of interpretable points. Moreover, physicians should consider previous published studies with caution, emphasizing the importance of sharing study data in order to reanalyze data by means of new methods.^[18]^ Finally, a more rigorous approach would be to exclude uninterpretable changes from the analysis, but if a study presents an excessively high proportion of uninterpretable changes, these changes could be reinterpreted by a complementary analysis. The degree of overlap between reference values (+/− RM × RC) of the change might be used to estimate the uncertainly of the change. Statistical weighting could be applied to the pair of 2 successive measurements in order to evaluate the probability that the measurement is interchangeable, in the gray zone or noninterchangeable. Therefore, the smaller the degree of overlap, the greater the weight that must be attributed to the pair of measurements. This method could be more hazardous than the use of noninterpretable changes, but it could allow complementary analysis of uninterpretable changes.

Certain limitations of the study must be addressed. First, we used a fixed repeatability coefficient for the classification of each change. This method supposes that repeatability remained constant over time and regardless of the range of values. Further studies could be conducted to address this issue. Second, the proposed trend interchangeability rate, classified as excellent, good, poor, or not clinically relevant in our study, was subjective, and may be open to criticism. Third, this new method must be validated in large-scale comparison studies.

## 5 Conclusions

None of pulse contour CO technique could be interchangeable with bolus thermodilution to assess changes in CO using the trend interchangeability method in cardiac surgery patients. Trend interchangeability could be a simple, objective method to compare changes of physiological or laboratory measurements obtained by different methods. Depending on the repeatability of the RM, the trend interchangeability rate can be used to define the proportion of interchangeable changes between different devices. Future studies should consider using the trend interchangeability rate method to assess the interchangeability of changes in measurements.

## Supplementary Material

Supplemental Digital Content

## Supplementary Material

Supplemental Digital Content

## Supplementary Material

Supplemental Digital Content
