# Supplementary material for: Evaluation of cardiac output by 5 arterial pulse contour techniques using trend interchangeability method
Source: Medicine (Baltimore). 2016 Jun 24;95(25):e3530. doi: 10.1097/MD.0000000000003530 (PMC4998299; doi:10.1097/MD.0000000000003530)
Supplement: Supplemental Digital Content [file medi-95-e3530-s001.pdf]

```
library(mvtnorm)
sigmaa=matrix(c(1.1,1,1,1.1), ncol=2)
z1=rmvnorm(n=150,mean=c(2.5,2.5),sigma=sigmaa)

sigma2=matrix(c(1,0.85,0.85,1), ncol=2)
z2=rmvnorm(n=150,mean=c(3.5,3.5),sigma=sigma2)
sigma3=matrix(c(1,0.75,0.75,1), ncol=2)
z3=rmvnorm(n=150,mean=c(5,5),sigma=sigma3)

C1=cbind(z1[,1],z2[,1],z3[,1])
C1[1:3,]
C2=cbind(z1[,2],z2[,2],z3[,2])
r1=matrix(t(C1),ncol=1)
r2=matrix(t(C2),ncol=1)
ID3=matrix(NA,ncol=3,nrow=nrow(z1))
for (i in 1:nrow(z1))
{
for (j in 1:3)
{
ID3[i,j]=i
}
}
ID=matrix(t(ID3),ncol=1)
BD=cbind(ID,r1,r2)
```
