# Supplementary figures and images for: Evaluation of cardiac output by 5 arterial pulse contour techniques using trend interchangeability method
Source: Medicine (Baltimore). 2016 Jun 24;95(25):e3530. doi: 10.1097/MD.0000000000003530 (PMC4998299; doi:10.1097/MD.0000000000003530)

## Slide 1
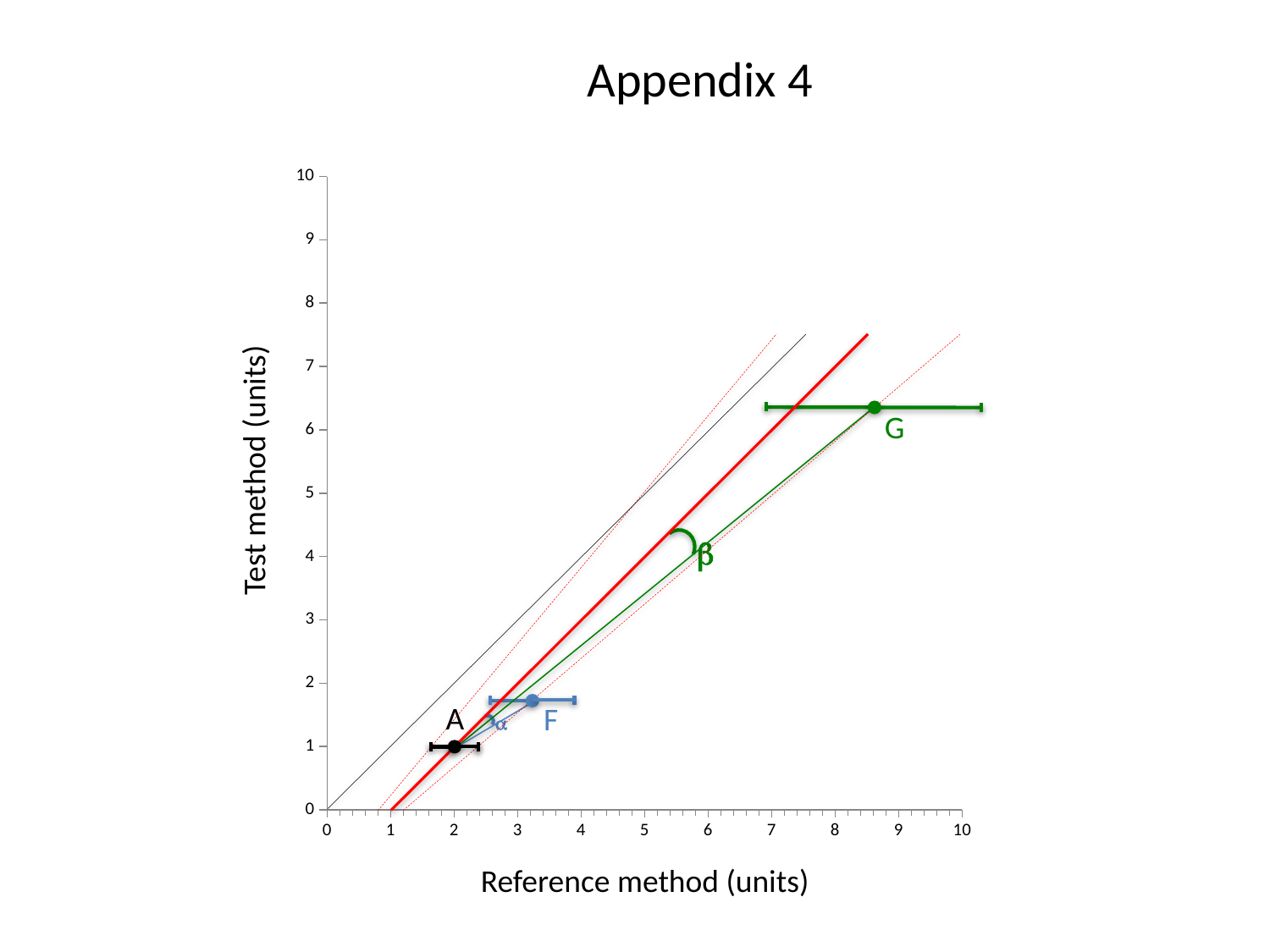

Appendix 4
### Chart
| Category | |
|---|---|G
Test method (units)
A
F
Reference method (units)

Supplement: Supplemental Digital Content [file medi-95-e3530-s003.pptx]
